# Internal hernia through the mesenteric defect in twin pregnancy: a case report and literature review

**DOI:** 10.3389/fmed.2024.1479634

**Published:** 2024-11-29

**Authors:** Lan Wang, Yuchun Zhu, Huayun Tan

**Affiliations:** Department of Obstetrics, Weifang People’s Hospital, Shandong Second Medical University, Weifang, China

**Keywords:** internal hernia, mesenteric defect, twin pregnancy, bowel obstruction, cesarean section

## Abstract

**Background:**

Internal hernias through mesenteric defects are rare, particularly in the context of twin pregnancies, and can lead to severe complications such as bowel obstruction and strangulation. Early diagnosis is critical, yet challenging, due to the overlapping symptoms with other abdominal conditions and the limited use of advanced imaging during pregnancy.

**Case description:**

We present a 33-year-old woman with a twin pregnancy at 33 + 2 weeks of gestation who experienced acute bowel obstruction due to an internal hernia through a congenital mesenteric defect. The patient presented with persistent upper abdominal pain, nausea, and vomiting. Given the advanced stage of pregnancy and the associated risks, a cesarean section was performed, followed by surgical exploration. Approximately one meter of strangulated small intestine was resected, and the mesenteric defect was repaired. Both mother and infants recovered uneventfully.

**Conclusion:**

This case highlights the importance of considering internal hernia in the differential diagnosis of acute abdominal pain during pregnancy. Prompt surgical intervention is crucial to prevent maternal and fetal morbidity.

## Introduction

Mesenteric defect hernias are a rare type of internal hernia that can lead to bowel obstruction and strangulation (1). These hernias can be congenital, resulting from developmental anomalies where the visceral and parietal peritoneum fail to fuse completely, or acquired, often related to previous abdominal surgeries. The increased intra-abdominal pressure during pregnancy can exacerbate the risk of herniation, making prompt diagnosis and treatment critical. This report presents a rare case of an internal hernia through a congenital mesenteric defect in a woman with a twin pregnancy and reviews the relevant literature. We present the following case by the CARE reporting checklist.

## Case presentation

A 33-year-old woman, G1P0A0, with a twin pregnancy at 33 + 2 weeks of gestation, presented with a 1-day history of persistent upper abdominal pain, nausea, and vomiting, and was admitted on May 30, 2023. She denied any history of trauma, major surgeries, hereditary diseases, or infectious diseases in her family. Her last menstrual period was on October 15, 2022, and she had undergone *in vitro* fertilization and embryo transfer (IVF-ET) on October 28, 2022. There were no significant genetic or psychosocial factors identified that could have contributed to this case, and her pregnancy had been otherwise uneventful until the onset of symptoms.

**FIGURE 1:**
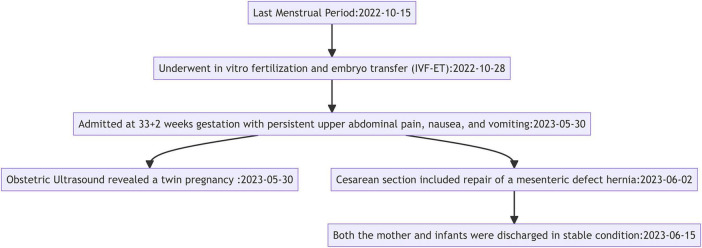
Clinical timeline for twin pregnancy with mesenteric hernia.

The timeline provides a concise overview of the patient’s journey from her last menstrual period to the successful management of her internal hernia, highlighting key clinical interventions that were critical to safeguarding the health of both the mother and her twins (Figure 1).

Physical examination revealed a temperature of 36.9°C, a pulse of 90 beats per minute, and a blood pressure of 139/89 mmHg. Her abdomen was distended with diminished bowel sounds. Initial laboratory results indicated an elevated white blood cell count (11.62 × 10^9/L; normal range: 3.5–9.5 × 10^9/L) with 91.6% neutrophils (normal range: 40–75%), normal amylase levels (44 U/L; normal range: 35–135 U/L), and an elevated D-dimer (9.21 μg/mL; normal range: 0.00–1.00 μg/mL).

An obstetric ultrasound on May 30, 2023, revealed a twin pregnancy: F1 in left occiput anterior (LOA) position (corresponding to 32 weeks and 3 days of gestation) and F2 in breech position (corresponding to 33 weeks and 4 days of gestation). She was treated with acid suppressants, gastric protectants, fluids, anti-inflammatory agents, and fetal lung maturation therapy. Despite these initial treatments, her symptoms persisted, and she developed a fever (38.0°C), leading to a clinical suspicion of acute gastroenteritis. In light of her presentation and pregnancy status, differential diagnoses considered included gastroenteritis, pancreatitis, acute bowel obstruction, and other potential causes of abdominal pain and inflammation during pregnancy, such as cholelithiasis, appendicitis, and urolithiasis. The challenges posed by limited imaging options due to pregnancy necessitated a careful and systematic approach to ruling out these conditions.

Due to the patient’s advanced stage of pregnancy, imaging studies like CT scans posed a potential risk to the fetus. Therefore, we opted for ultrasound and laboratory tests to evaluate the patient’s condition. The laboratory results showed an elevated white blood cell count (11.62 × 10^9/L; normal range: 3.5–9.5 × 10^9/L) with 91.6% neutrophils (normal range: 40–75%), indicating a high likelihood of acute inflammation or infection. Additionally, D-dimer levels were significantly elevated (9.21 μg/mL; normal range: 0.00–1.00 μg/mL), suggesting the possibility of a thrombotic event or other acute pathology. Despite administering 30 mL of oral lactulose, the patient still did not pass gas or stool, and continued to experience nausea, vomiting, and an inability to eat, further raising the suspicion of acute bowel obstruction. The patient also reported unbearable abdominal distension and declined conservative treatment. Given these considerations, we ultimately decided to proceed with a cesarean section followed by surgical exploration. We invited the surgical team for an intraoperative consultation and informed the neonatology department to prepare for potential adverse outcomes in the fetus.

**FIGURE 2:**
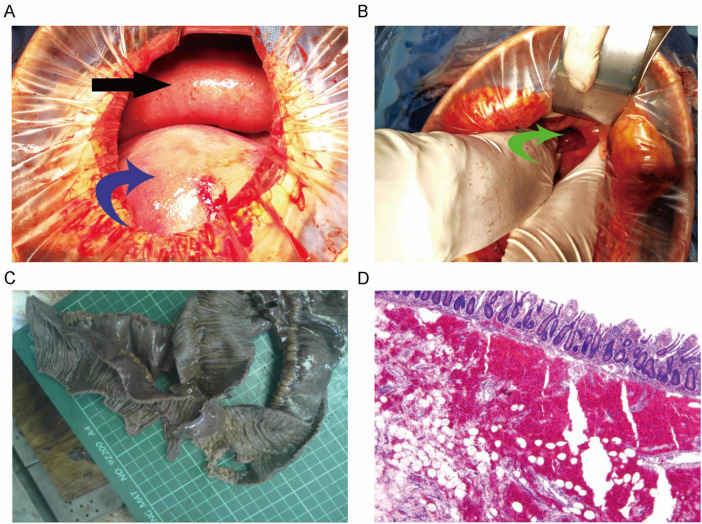
Surgical and pathological findings of internal hernia in twin pregnancy. **(A)** Intraoperative view showing the congested and edematous small intestine (black arrow) and the gravid uterus (blue arrow). **(B)** Terminal ileum incarcerated in a mesenteric defect (green arrow). **(C)** Resected segment of necrotic bowel showing darkened. **(D)** Histological section of the resected bowel demonstrating acute and chronic inflammation, vascular congestion, hemorrhage, and edema in the submucosal layer (magnification ×40).

During the surgery on June 2, 2023, approximately 200 mL of dark red ascitic fluid was noted, and significantly distended and edematous bowel loops were observed above the uterus (Figure 2A). Two female infants were delivered with Apgar scores of 10 at 1 min, and they were transferred to the neonatal intensive care unit. After suturing the uterus, surgical exploration revealed terminal ileum incarcerated in a small bowel mesenteric defect (Figure 2B). About 1 meter of the small intestine was strangulated. The herniated bowel was reduced, and a darkened segment, suspected to be necrotic, was resected (Figure 2C). The hernia was eliminated after suturing the mesenteric defect and performing an end-to-end anastomosis to restore bowel continuity. Histopathological examination of the resected bowel showed acute and chronic inflammation of the mucosa, vascular congestion, hemorrhage, and edema throughout the bowel wall (Figure 2D).

The patient was discharged in stable condition on June 15, 2023, and subsequent follow-up visits, including the last follow-up on August 1, 2024, showed no further complications. The patient’s bowel function has returned to normal without signs of recurrence. Both the mother and infants are in good health, and long-term monitoring will continue to ensure there are no residual issues from the internal hernia or surgical interventions.

## Discussion

Hernias during pregnancy can present in various forms, each with unique etiologies and clinical implications. The increased intra-abdominal pressure from the growing uterus predisposes pregnant women to hernias, including diaphragmatic hernias, Spigelian hernias, Petersen’s hernias, and internal hernias through mesenteric defects (1–10). Table 1 summarizes relevant case reports and studies on internal hernias in pregnant women. These conditions can be congenital or acquired, often exacerbated by previous surgeries such as bariatric procedures or trauma.

**TABLE 1:** Summary of hernia in pregnancy in the literature.

References	Age	Gestational week	Clinical presentation and cause of hernia in pregnancy	Intraoperative findings and treatment	Outcome
Yuansheng et al. (1)	31	16 weeks	Nausea, vomiting, left upper abdominal pain; congenital transmesenteric defect	Emergency laparotomy, defect repair, resection of necrotic bowel	Good recovery for both mother and baby
Haj-Yahia et al. (2)	29	Full term	Shock, acute abdominal pain; diaphragmatic rupture following CPR	Emergency laparotomy, diaphragmatic defect repair	Good recovery, no complications
Borghede et al. (3)	22	22 weeks	Abdominal pain, vomiting, diarrhea; Internal hernia post bariatric surgery	Surgical intervention, bowel resection	Good recovery
Calderón Espinosa de Los Monteros et al. (4)	42	34 weeks	Abdominal pain, vomiting; Petersen’s hernia post RYGB	Emergency surgery	Good recovery
Kassir et al. (5)	30	28 weeks	Abdominal pain, swelling; Spigelian hernia due to necrobiotic fibroma	Medical management	Good recovery, no complications
Joyeux et al. (6)	33	32 weeks	Abdominal pain, nausea, vomiting, bowel obstruction symptoms; Intestinal malrotation	Emergency C-section and laparotomy, defect repair	Good recovery for both mother and baby
Warsza et al. (7)	35	30 weeks	Abdominal pain, vomiting; internal hernia post RYGB	Emergency laparotomy, defect repair	Good recovery, no complications
Benson et al. (8)	23	20 weeks	Left upper quadrant pain; diaphragmatic hernia related to abandoned epicardial pacemaker wires	Emergency thoracic surgery	Good recovery, no complications
Chuzi et al. (9)	26	20 weeks	Shortness of breath, left-sided chest pain, nausea, vomiting; left diaphragmatic rupture	Emergency thoracic surgery	Good recovery, no complications
Kannan et al. (10)	30	21 weeks	Upper abdominal pain, nausea, vomiting; internal hernia post RYGB	Emergency laparoscopic surgery, defect repair	Good recovery, delivered healthy baby at term

Typically, mesenteric defects arise from previous abdominal surgeries or traumatic injuries (1). However, in our patient’s case, there was no history of trauma or prior surgeries. Therefore, it was determined that the mesenteric defect was congenital in nature (11).

Congenital mesenteric defects, potentially caused by inadequate degeneration of the dorsal mesentery, rapid growth of mesenteric segments, and expansion of low blood supply areas during development, are rare but significant causes of intestinal obstruction. Without prompt diagnosis and treatment, these defects can lead to severe complications like small bowel necrosis (11).

Diagnosing internal hernias is particularly challenging due to the lack of early radiographic findings and specific laboratory biomarkers. Advanced imaging techniques, such as CT scans, are instrumental in diagnosing internal hernias (6, 7). However, their use during pregnancy is often limited due to potential risks to the fetus. In our case, the advanced gestational age of 33 + 2 weeks, along with the history of IVF and twin pregnancy, presented unique challenges. While CT scans are typically crucial for diagnosing internal hernias, they were contraindicated due to potential fetal risks. MRI, although free of ionizing radiation, poses potential risks because gadolinium contrast can cross the placental barrier. Moreover, MRI does not clearly outperform CT in detecting internal hernia signs such as clustered loops, dilated and displaced bowel loops, or mesenteric edema (7).

This limitation in using advanced imaging technologies made diagnosis and treatment more complex. Due to inflammatory symptoms, CT was not performed, and differential diagnosis was based on excluding other acute abdominal diseases, including cholelithiasis, pancreatitis, urolithiasis, appendicitis, and colitis. Identifying the cause of small bowel obstruction was particularly challenging. Further questioning revealed that the patient had not passed gas or stool for 2 days.

Given the high fetal survival rate, we proceeded with a cesarean section to terminate the pregnancy and explore the cause of bowel obstruction. During surgery, an internal hernia was discovered, and a darkened bowel segment, suspected to be necrotic, was resected. Potentially fatal complications, including strangulated bowel obstruction, perforation, and death, were avoided. After successfully delivering two viable female infants and suturing the uterus, the non-viable bowel was resected, the hernia was eliminated, and the defect was repaired, thereby preventing maternal or fetal mortality.

In the face of diagnostic challenges, the importance of a multidisciplinary approach in managing this case cannot be overstated. The successful outcome was the result of close collaboration between the obstetric, surgical, and neonatal teams. The obstetric team played a key role in the timely recognition of the abdominal emergency, while the surgical team ensured prompt surgical exploration and treatment. This coordination was critical, especially given the challenges posed by limited diagnostic tools during pregnancy. Neonatologists were also involved early in the process to ensure the immediate care of the twins following delivery. This integrated approach allowed for rapid decision-making, minimizing delays, and avoiding severe complications such as bowel necrosis, perforation, and fetal or maternal mortality. In similar complex cases, a well-coordinated multidisciplinary team is essential for optimizing both maternal and fetal outcomes.

## Patient’s perspective

After a challenging pregnancy with twins, learning I had a rare congenital mesenteric hernia was one more hurdle. I’m incredibly thankful for the quick and coordinated response from the obstetrics team and the surgical department. Their immediate actions and teamwork guaranteed that my twins were born healthy and that I stayed out of danger. I’m profoundly grateful.

## Conclusion

This case highlights the importance of considering internal hernias as a differential diagnosis in pregnant women presenting with acute abdominal pain, especially in multifetal gestations. The congenital mesenteric defect, undetected until late pregnancy, underscores the need for vigilance when imaging options are limited. Prompt surgical intervention was key to the favorable outcome, ensuring both maternal and neonatal safety. This case reinforces the critical need for timely diagnosis and intervention in similar clinical scenarios to prevent severe complications.

## Data Availability

All relevant data related to this case report are presented within the article. For additional information, please contact the corresponding author.
